# Synthesis of New Derivatives of Berberine Canagliflozin and Study of Their Antibacterial Activity and Mechanism

**DOI:** 10.3390/molecules29010273

**Published:** 2024-01-04

**Authors:** Jinsheng Li, Xueli Hou, Jinlong Xiao, Li Zhu, Yujie Deng, Ziyi Li, Zijian Zhao, Zhenghong Luo, Hao Wei

**Affiliations:** 1 Department of Pharmacology, Shaanxi University of Chinese Medicine, Xianyang 712046, China; jsli1006@163.com (J.L.); xlhou199885@163.com (X.H.); weihao217@163.com (H.W.); 2 School of Chemistry and Materials Science, Huaihua University, Huaihua 418000, China; jlxiao0204@163.com (J.X.); lizzz082803@163.com (L.Z.); dyj20040128@163.com (Y.D.); zyli1202@163.com (Z.L.); 3 Key Laboratory of Research and Utilization of Ethnomedicinal Plant Resources of Hunan Province, Huaihua University, Huaihua 418000, China

**Keywords:** berberine, canagliflozin, antibacterial activity

## Abstract

The isoquinoline alkaloid berberine, derived from Coptidis rhizoma, exhibits antibacterial, hypoglycemic, and anti-inflammatory properties. Canagliflozin is a sodium–glucose cotransporter 2 (SGLT2) inhibitor. We synthesized compounds B9OC and B9OBU by conjugating canagliflozin and n-butane at the C9 position of berberine, aiming to develop antimicrobial agents for combating bacterial infections worldwide. We utilized clinically prevalent pathogenic bacteria, namely *Staphylococcus aureus*, *Escherichia coli*, and *Pseudomonas aeruginosa*, to investigate the antibacterial efficacy of B9OC. This was accomplished through the determination of the MIC_80_ values, analysis of bacterial growth curves, evaluation of biofilm formation using crystal violet staining, assessment of impact on bacterial proteins via SDS-PAGE analysis, and observation of alterations in bacterial morphology utilizing field emission scanning electron microscopy. Meanwhile, the ADMET of compound B9OC was predicted using a computer-aided method. The findings revealed that B9OC exhibited lower minimal inhibitory concentrations against all three bacteria compared to berberine alone or in combination with canagliflozin. The minimal inhibitory concentrations (MICs) of B9OC against the three experimental strains were determined to be 0.035, 0.258, and 0.331 mM. However, B9OBu exhibited a lower level of antimicrobial activity compared to berberine. The compound B9OC exhibits a broad spectrum of antibacterial activity by disrupting the integrity of bacterial cell walls, leading to cellular rupture and the subsequent degradation of intracellular proteins.

## 1. Introduction

**Figure 1:**
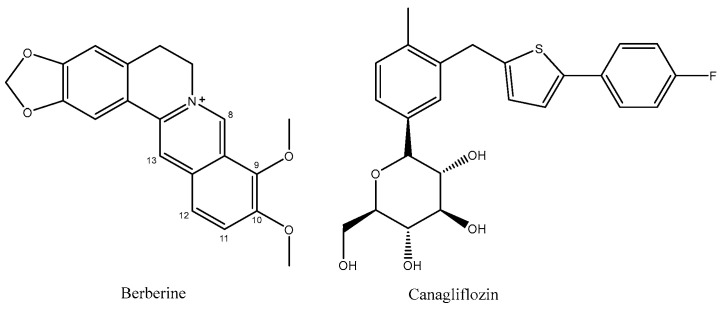
Structures of berberine and canagliflozin.

**Figure 2:**
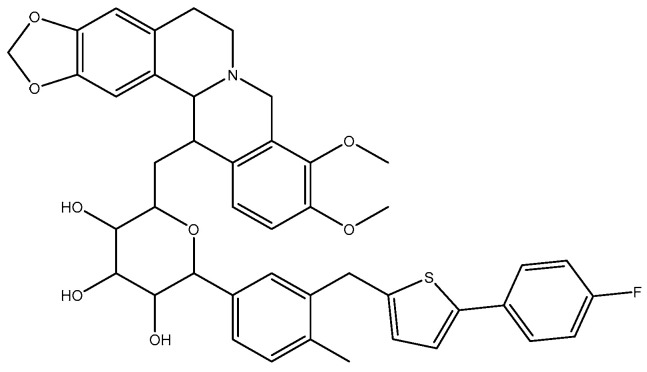
Structures of derivative, BC.

**Figure 3:**
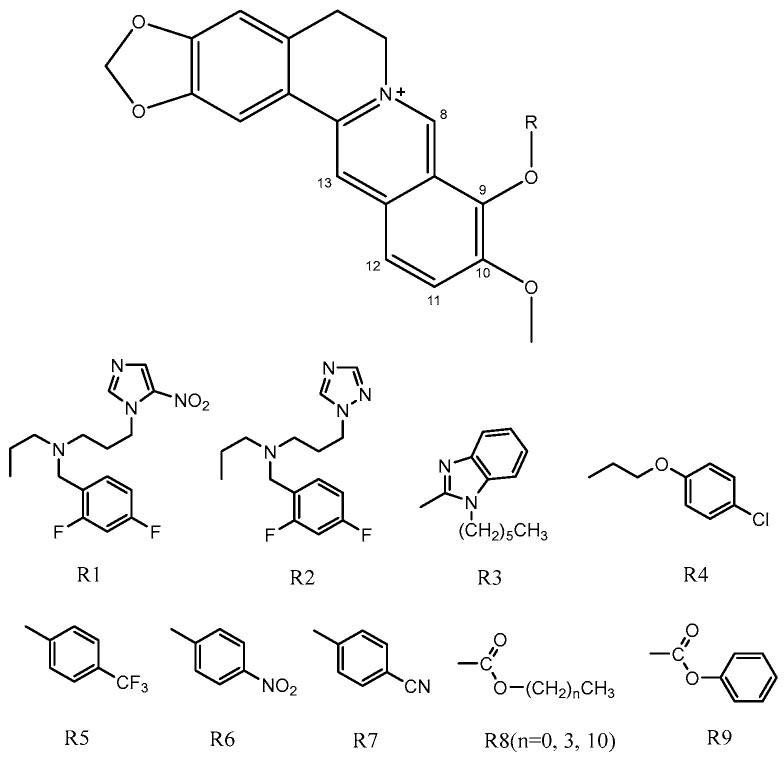
Structures of berberine derivative with antimicrobial activity at position C9.

Berberine (BBR, C_20_H_18_NO_4_, MW 336.37, Figure 1), a natural isoquinoline alkaloid, is a pivotal component in numerous traditional Chinese medicines. It can be derived from plants such as the three-leaf Coptis of the Berberidaceae family, *Huanglian* of the Ranunculaceae family, and *Huangbo* of the Rutaceae family [1]. Commonly encountered as yellow needle-shaped crystals, berberine hydrochloride represents a salt form widely utilized as an oral broad-spectrum antibiotic for gastrointestinal infections. Moreover, its heat-clearing and detoxifying properties have been extensively harnessed over prolonged durations. With its cost-effectiveness and well-established safety and efficacy profile, berberine hydrochloride emerges as an invaluable naturally derived extract in traditional medicine. The extensive pharmacological activities of berberine are attributed to its potent antibacterial and antiviral properties, as well as its ability to regulate blood glucose levels, protect cardiovascular health, modulate metabolism, and exhibit anti-cancer effects. Consequently, it finds application in the treatment of various diseases [2,3,4,5]. Canagliflozin (CAN, C_24_H_25_FO_5_S, MW 444.52, Figure 1) is a sodium–glucose co-transporter 2 (SGLT2) inhibitor commonly used in the treatment of diabetes. Studies in the literature have demonstrated that the combination of berberine (BBR) and canagliflozin (CAN) exhibits synergistic effects in reducing hyperglycemia associated with diabetes while minimizing adverse effects [6]. Therefore, in our previous study, we synthesized a novel compound BC (Figure 2) by chemically linking canagliflozin at the C13 position of berberine to enhance its hypoglycemic efficacy. Surprisingly, the results revealed that compound BC did not exhibit significant hypoglycemic activity; however, it displayed potent antibacterial properties against *Pseudomonas aeruginosa* [7]. Currently, approximately 700,000 individuals succumb to bacterial infections annually worldwide. Based on the prevailing trajectory, it is projected that by 2050, around 10 million people will perish due to superbug infections [8]. Concurrently, there is a continuous rise in bacterial resistance towards antibiotics [9]. Consequently, the urgent imperative within the global healthcare domain lies in the discovery of novel antibiotics capable of combating superbugs. Recently conducted studies on the antibacterial activity of berberine derivatives have revealed that modifications at positions C8, 9, and 12, in addition to position C13, can significantly enhance the efficacy of berberine against bacteria. Based on our comprehensive understanding of the properties of both berberine and canagliflozin, as well as extensive literature research, we propose a hypothesis that conjugating canagliflozin at position C9 of berberine may result in a compound exhibiting remarkably potentiated antibacterial effects (see Figure 3) [10,11,12,13]. Building upon this premise, we have devised and synthesized a derivative of berberine known as B9OC, with an aim to explore its antibacterial activity and underlying mechanism.

## 2. Results and Discussion

### 2.1. Analysis of Berberrubine

Berberrubine: red solid, ^1^H NMR (400 MHz, Chloroform-*d*) δ 9.18 (s, 1H), 7.56 (s, 1H), 7.24 (d, *J =* 7.5 Hz, 2H), 6.75 (s, 1H), 6.48 (d, *J* = 7.9 Hz, 1H), 6.06 (s, 2H), 4.40 (t, *J* = 6.1 Hz, 2H), 3.89 (s, 3H), 3.08 (t, *J* = 6.1 Hz, 2H), and 2.44 (s, 1H).^13^C NMR (101 MHz, Chloroform-*d*) δ 150.83, 149.07, 148.14, 145.77, 132.98, 131.36, 128.22, 122.22, 120.44, 120.09, 117.59, 108.41, 104.56, 102.97, 101.86, 77.36, 56.09, 53.33, and 28.65.

### 2.2. Analysis of Br-C

Canagliflozin bromide: white solid, ^1^H NMR (400 MHz, Chloroform-*d*) δ 7.46–7.38 (m, 2H), 7.25–7.11 (m, 3H), 7.08–6.92 (m, 3H), 6.63 (d, *J* = 3.6 Hz, 1H), 5.30 (s, 2H), 4.43 (s, 1H), 4.15–4.07 (m, 3H), 4.02 (d, *J* = 12.4 Hz, 1H), 3.69–3.56 (m, 4H), 3.44 (dd, *J* = 8.7, 3.3 Hz, 2H), 3.07 (d, *J* = 3.7 Hz, 1H), and 2.27 (s, 3H). ^13^C NMR (101 MHz, Chloroform-*d*) δ 143.05, 141.68, 138.57, 137.12, 135.95, 130.97, 130.81, 128.82, 127.26, 127.18, 126.18, 125.85, 122.85, 115.96, 115.74, 81.44, 77.72, 77.36, 75.32, 71.94, 53.58, 34.25, 33.65, and 19.43.

### 2.3. Analysis of B9OC

9-berberrubine-(9→6′)-*O*-canagliflozin derivative: yellow sloid, ^1^H NMR (400 MHz, Chloroform-*d*) δ 7.14 (s, 1H), 6.76 (d, *J* = 8.2 Hz, 1H), 6.59 (d, *J* = 8.4 Hz, 2H), 5.99–5.88 (m, 3H), 5.75 (s, 1H), 5.35–5.27 (m, 2H), 3.87 (s, 3H), 3.71–3.59 (m, 1H), 3.48 (dt, *J* = 11.1, 4.9 Hz, 1H), 3.01–2.71 (m, 3H), and 2.45 (dd, *J* = 16.6, 4.6 Hz, 1H). ^13^C NMR (101 MHz, Chloroform-*d*) δ 165.49, 150.39, 148.69, 147.63, 144.40, 140.43, 137.60, 134.72, 130.62, 129.64, 128.95, 127.47, 123.58, 119.17, 115.43, 114.91, 110.89, 108.11, 108.06, 104.89, 104.38, 103.71, 101.63, 101.22, 95.30, 77.36, 56.84, 56.42, 55.09, 47.95, 39.25, 30.62, 29.84, 28.54, and 18.61. MS spectrum (TOF MS) *m*/*z* (%): 748.3185 (calcd. for C_43_H_39_FNO_8_S, 748.2375). The purity of BC was identified as about 97.257% by HPLC at 365 nm (Supplementary Materials).

### 2.4. Analysis of B9OBU

Berberine 9 oxybutyl derivative: yellow sloid, ^1^H NMR (400 MHz, DMSO-*d*_6_) δ 10.29 (s, 1H), 8.36 (s, 1H), 7.91 (d, *J* = 9.0 Hz, 1H), 7.74 (d, *J* = 9.0 Hz, 1H), 7.36 (s, 1H), 6.77 (s, 1H), 6.04 (s, 2H), 5.32 (t, *J* = 6.3 Hz, 2H), 4.45 (t, *J* = 6.8 Hz, 2H), 4.01 (s, 3H), 3.31 (t, *J* = 6.3 Hz, 2H), 1.99 (p, *J* = 6.9 Hz, 3H), 1.57 (q, *J* = 7.5 Hz, 3H), 1.24 (s, 3H), and 1.01 (t, *J* = 7.4 Hz, 4H). ^13^C NMR (101 MHz, Chloroform-*d*) δ 150.75, 148.39, 146.76, 137.68, 133.48, 130.62, 126.11, 123.02, 122.49, 120.38, 119.85, 108.61, 105.42, 102.22, 77.36, 75.46, 57.09, 56.21, 32.33, 29.83, 27.79, 19.22, and 14.09.

### 2.5. MIC_80_ of B9OC, BBR, CAN, B + C, and B9OBU against E. coli, S. aureus, and P. aeruginosa

The compounds B9OC, CAN, BBR, BBR + CAN, and B9OBU all demonstrate antibacterial activity against *S. aureus*, *E. coli*, and *P. aeruginosa* (refer to Table 1). The minimum inhibitory concentrations (MICs) of B9OC against *S. aureus*, *E. coli*, and *P. aeruginosa* are 0.035 mM, 0.285 mM, and 0.331 mM, respectively. The MIC_80_ values of BBR against the three bacteria are determined as 0.188 mM for *S. aureus*, 0.982 mM for *E. coli*, and 0.665 mM for *P. aeruginosa*. In the case of combination treatment with BBR + CAN, the MIC_80_ values against the three bacteria are found to be at concentrations of 0.063 mM, 0.435 mM, and 0.251 mM, respectively. The MIC_80_ values of B9OBU against the three bacteria are observed at concentrations of 0.591 mM, 2.241 mM, and 1.283 mM, respectively. CAN did not exhibit any detectable MIC_80_ value against *P. aeruginosa* at experimental concentrations (<5.12 mM). In comparison to other drugs tested in the experiment, compound B9OC demonstrates superior antibacterial efficacy and possesses broad-spectrum antimicrobial activity. It exhibits inhibitory effects on both Gram-positive and Gram-negative bacteria. Our previous studies indicated that compound BC, synthesized by our team, exhibited MIC_80_ values of 0.38, 0.39, and 0.22 mM against three bacterial strains [4]. Compared to BC, B9OC displays a lower minimum inhibitory concentration against *S. aureus* and *E. coli*. Interestingly, introducing a functional group at position 9 of berberine (B9OBU) actually diminishes its antibacterial effectiveness. Moreover, the combination of berberine (BBR) and canagliflozin (CAN) demonstrates enhanced antibacterial efficacy as opposed to using BBR alone.

### 2.6. Effects of B9OC on E. Coli, S. aureus, and P. aeruginosa Growth

**Figure 4:**
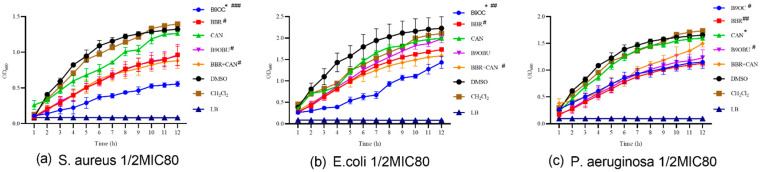
Effects of B9OC, BBR, CAN, B9OBU, and BBR + CAN on the growth of *Staphylococcus aureus*, *Escherichia coli*, and *Pseudomonas aeruginosa*. Responses of (**a**) *Staphylococcus aureus*, (**b**) *Escherichia coli*, and (**c**) *Pseudomonas aeruginosa* to B9OC, BBR, CAN, B9OBU, and BBR + CAN at 1/2MIC_80_. * *p* < 0.05; ^#^ *p* < 0.05, ^##^ *p* < 0.01, ^###^ *p* < 0.001 vs. Ctrl (DMSO or CH_2_Cl_2_). Data are shown as mean ± SD (*n* = 3).

The effects of B9OC, BBR, CAN, B9OBU, and BBR + CAN on the growth of *S. aureus*, *E. coli*, and *P. aeruginosa* were evaluated (refer to Figure 4). Among these drugs, B9OC, BBR, and B9OBU demonstrated significant inhibitory effects on the growth of all three bacterial strains. Notably, B9OC exhibited the strongest bacteriostatic effect. Compared to BBR, drug B9OC displayed a more potent inhibition against the growth of *S. aureus* and *E. coli*. No significant differences were observed in the effects of either BBR or its combination with CAN (BBR + CAN) on the growth of these bacterial strains. Furthermore, CAN did not exert any notable impact on the growth of any experimental bacteria strains.

### 2.7. Antibiofilm Activity of B9OC

**Figure 5:**
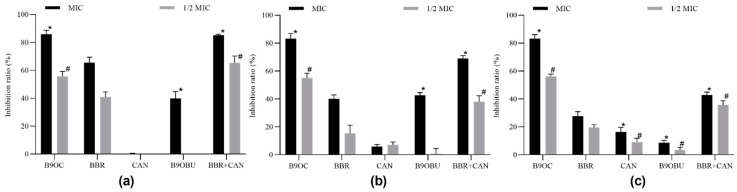
Antimicrobial effects of B9OC, BBR, CAN, BBR + CAN, and B9OBU. The bacterial strain: (**a**) *Staphylococcus aureus*, (**b**) *Escherichia coli*, and (**c**) *Pseudomonas aeruginosa*. Data are expressed as mean ± SD (*n* = 3); * *p* < 0.05 vs. BBR(MIC); ^#^ *p* < 0.05 vs. BBR(1/2MIC).

**Figure 6:**
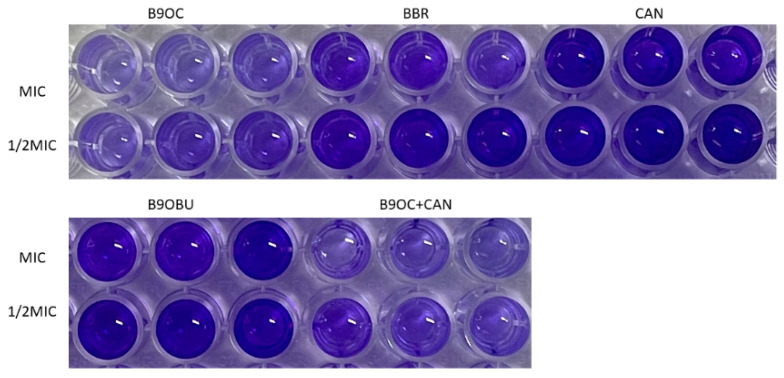
Biofilm formation of *Staphylococcus aureus*.

The anti-biofilm efficacy of B9OC was investigated on three test strains using the crystal violet staining method (Figure 5 and Figure 6). B9OC, BBR, and BBR + CAN exhibited inhibitory effects on biofilm formation of all three test strains at MIC and 1/2MIC concentrations. Among them, B9OC demonstrated the most potent inhibitory effect. The combination of BBR + CAN showed a stronger impact on bacterial biofilms compared to that of BBR alone. CAN had no significant effect on *S. aureus* biofilms, while its influence on *E. coli* and *P. aeruginosa* biofilms was weaker. At 1/2MIC concentration, B9OBU did not exhibit a significant effect on *S. aureus* and *E. coli* biofilms. Figure 5 visually presents the crystal violet staining results of biofilms adhered to the pore walls, highlighting that B9OC has the greatest impact on *S. aureus* biofilms. Therefore, it can be inferred that the antibacterial mechanism of action for B9OC is associated with disrupting bacterial biofilms.

### 2.8. Sds-Page Analysis

**Figure 7:**
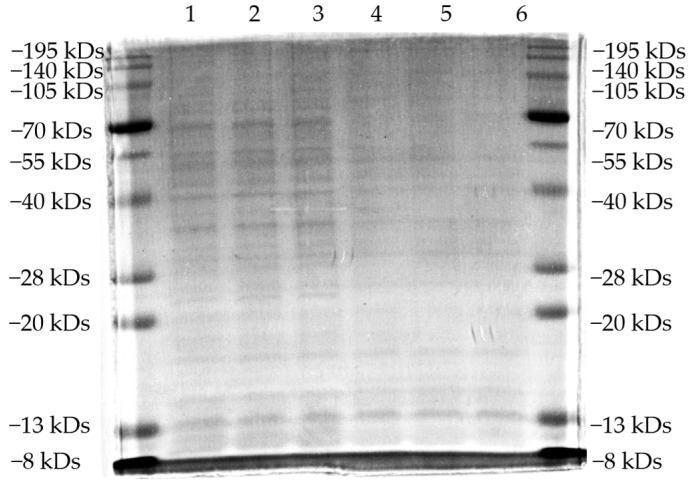
SDS-PAGE analysis of intracellular soluble proteins of *Staphylococcus aureus* treated with 1/2MIC B9OC for 24 h. 1−3: Untreated *Staphylococcus aureus*; 4−6: *Staphylococcus aureus* after 24 h treatment with 1/2MIC B9OC.

**Figure 8:**
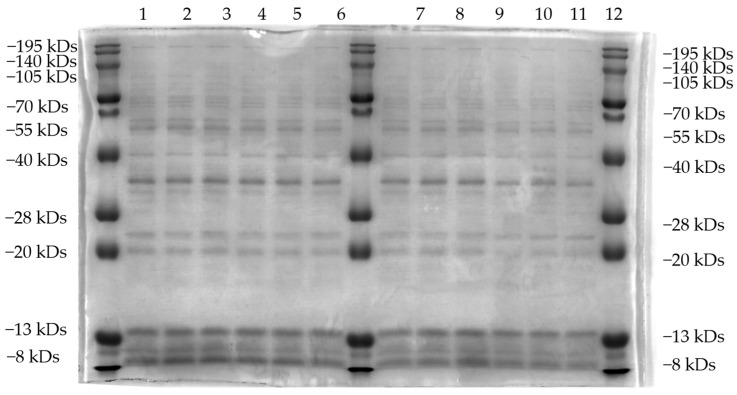
SDS-PAGE analysis of intracellular soluble proteins of *Staphylococcus aureus* treated with 1/2MIC BBR, CAN, and BBR + CAN for 24 h. 1−3: Untreated *Staphylococcus aureus*; 4−6: *Staphylococcus aureus* after 24 h treatment with 1/2MIC BBR; 7−9: *Staphylococcus aureus* after 24 h treatment with 1/2MIC CAN 10−12: *Staphylococcus aureus* after 24 h treatment with 1/2MIC BBR + CAN.

The effects of B9OC, BBR, CAN, and BBR + CAN on the protein levels of *Staphylococcus aureus* were analyzed using SDS-PAGE (Figure 7 and Figure 8). It can be observed that the protein bands of *Staphylococcus aureus* treated with B9OC exhibited a noticeable decrease in intensity. Therefore, it can be inferred that B9OC exerts a detrimental effect on the proteins within *Staphylococcus aureus*. The changes in protein bands after treatment with BBR and CAN were not statistically significant. Although the protein bands slightly decreased in intensity after treatment with BBR + CAN, it was not as pronounced as observed with B9OC. Based on the analysis of the experimental results, it can be concluded that the antibacterial mechanism of action for B9OC is associated with its disruption of bacterial proteins.

### 2.9. The Morphology of Bacteria Observed by FESEM

**Figure 9:**
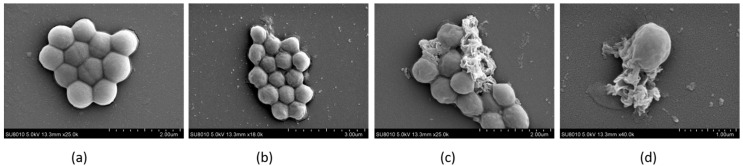
Field emission scanning electron micrographs of *Staphylococcus aureus*. (**a**) Untreated control cells at 24 h post-inoculation. (**b**–**d**) Bacterial cells treated with B9OC at 1/2MIC_80_ for 24 h.

The morphology of the bacteria was examined using field emission scanning electron microscopy (FESEM), as depicted in Figure 9. It is evident that the untreated bacterial cells exhibited a smooth and intact surface devoid of any wrinkles or grooves. In contrast, the B9OC-treated bacteria displayed pronounced wrinkling and indentation on their cellular surfaces, ultimately leading to cellular rupture. This observation strongly suggests that B9OC inflicted damage upon the bacterial cell wall, which aligns with the findings obtained from Section 2.7 and Section 2.8.

### 2.10. ADMET of B9OC

The ADMET results of compound B9OC, as predicted by computer-aided methods, are presented in Table 2. From the prediction results, the Human Intestinal Absorption (HIA) Probability of compound B9OC is 0.15 and the Oral Bioavailability (human) is 0.47; Plasma Protein Binding (human) is 0.97, Blood–Brain Barrier Permeability (BBBP) Probability is 0.26, and the Blood–Brain Ratio is −0.62; HLM-CLint (μL/min/mg) is 67.21, MLM-CLint (μL/min/mg) is 415.94, and RLM-CLint (μL/min/mg) is 89.628; CYP Induction Probability is 0.269 and average CYP Inhibition Probability is 0.732; hERG Inhibition Probability_cls10 is 0.614 and hERG Inhibition Probability_cls50 is 0.998; Hek293 Toxicity Probability is 0.952 and Hepatic Toxicity Probability is 0.967; Log(LD50) is 2.915; Tubulin Inhibition is 1; DILI (drug-induced liver injury) is 0.928; Genotoxicity Probability is 0.99, Phospholipidosis is 0.77, and Reproductive Toxicity is 0.997.

## 3. Materials and Methods

### 3.1. Materials

Canagliflozin, manufactured by Shanghai Aladdin Biochemical Technology Co., Ltd. (Shanghai, China) Berberine, which is extracted from coptis root and identified through nuclear magnetic resonance (NMR) analysis, has been confirmed as berberine. This experiment employed protein peptone (Oxoid, Hampshire, UK), yeast extract (Oxoid, Hampshire, UK), crystal violet (Beyotime, Shanghai, China), the SDS-PAGE gel preparation kit (Beyotime, Shanghai, China), SDS-PAGE protein loading buffer (5×) (Beyotime, Shanghai, China), Coomassie brilliant blue ultra-fast staining solution (Beyotime, Shanghai, China), pre-stained protein marker VII (8–195 kDa) (Savier, Wuhan, China), and the BCA protein concentration determination kit (Beyotime, Shanghai, China). Unless otherwise specified, all other reagents used in the experiment were commercially purchased.

### 3.2. Synthesis of Instruments and Compounds

The product mixtures were analyzed using fluorescent indicator thin-layer chromatography glass backplate (TLC) provided by Yantai Jiangyou Silica gel Development Co., Ltd. (Yantai, China). The multifunctional microplate reader (WD-2102B) was obtained from Beijing Liuyi Biotechnology Co., Ltd. (Beijing, China). The WB exposure instrument (GelView 6000Plus) was sourced from Guangzhou Biolight Biotechnology Co., Ltd. (Guangzhou, China). Biorad (Hercules, CA, USA) supplied the electrophoresis apparatus and electroconversion device (1645050). Beijing Yataikelong (Beijing, China) provided the ultra-clean workbench (YT-CJ-1NB). UV-active compounds were detected using the WD-9403A UV instrument (Beijing, China) (λ = 254 nm, 365 nm). Silica gel with a particle size of 300 to 400 mesh served as the stationary phase for column chromatography. Deuterated reagent peaks were utilized as internal standards for ^1^H and ^13^C NMR analysis in deuterated chloroform (^1^H-NMRδ = 7.26ppm, ^13^C-NMRδ = 77.16 ppm) and deuterated DMSO (^1^H-NMRδ = 2.50 ppm, ^13^C-NMRδ = 39.96 ppm). Chemical shift values are expressed in parts per million (ppm), spin–spin coupling constants in Hertz (Hz), and multiplicity is abbreviated as s (singlet), d (doublet), t (triplet), q (quartet), and m (multiplet).

### 3.3. Synthesis of Berberrubine

**Figure 10:**
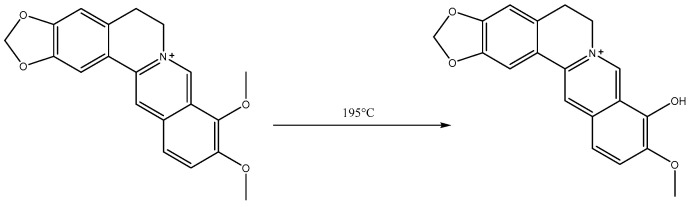
Synthesis of berberrubine.

The berberine was accurately weighed and transferred into a flask. Subsequently, the flask was introduced into a vacuum drying oven under a vacuum pressure of 20 to 30 mmHg and subjected to heating at 195 °C for a duration of 0.5–1 h. During this process, the yellow solid transformed into a dark red color and subsequently cooled down to room temperature [14,15,16]. Following that, the samples were dissolved in methanol and dichloromethane before being subjected to silica gel column chromatography (eluate: DCM:MeOH = 10:1). The resulting product was collected, yielding red solid berberrubine (Figure 10).

### 3.4. Synthesis of Canagliflozin Bromide (Br-C)

**Figure 11:**
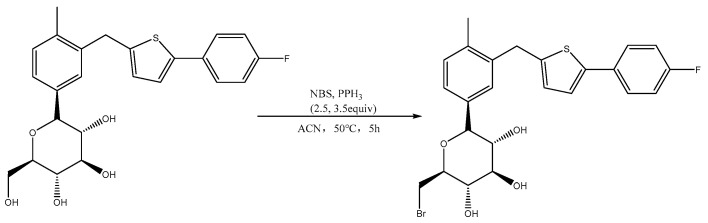
Synthesis of canagliflozin bromide (Br-C).

Canagliflozin (1 equivalent) was dissolved in anhydrous acetonitrile, followed by the addition of *N-Bromosuccinimide* NBS (2.5 equivalents) and *Triphenylphosphine* PPh_3_ (3.5 equivalents) under cooled conditions. The temperature was then raised to 50 °C and stirred for 5 h. After cooling to room temperature, the acetonitrile solvent was removed through spin evaporation, and the product was further purified using silica gel column chromatography with a mixture of DCM:MeOH = 40:1 as the eluent. Finally, a white foamy solid, canagliflozin bromide (Br-C), was obtained (Figure 11).

### 3.5. Synthesis of 9-Berberrubine-(9→6′)-O-canagliflozin Derivative (B9OC)

**Figure 12:**
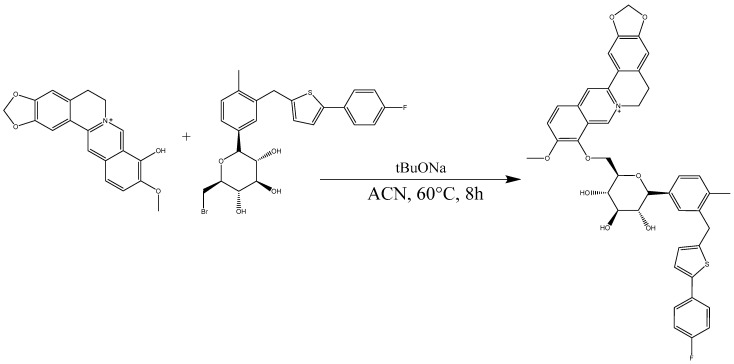
Synthesis of 9-berberrubine-(9→6′)-*O*-canagliflozin derivative (B9OC).

After consulting the synthetic methodologies employed by other researchers [17,18,19] without yielding any positive outcomes, we endeavored to modify the experimental conditions. Following numerous attempts, a suitable synthesis approach was eventually established. Berberrubine (1.2 equivalents), canagliflozin bromide (1 equivalent), and sodium tert-butyl alcohol (2 equivalents) were dissolved in 20 mL of anhydrous acetonitrile under argon protection, with the temperature set at 60 °C for stirring over an 8 h period. The solvent was subsequently removed via rotary evaporation. Purification was accomplished using dichloromethane as the eluent through silica gel column chromatography. This process led to the isolation of the yellow solid product known as 9-berberrubine-(9→6′)-*O*-canagliflozin derivative (B9OC) (Figure 12).

### 3.6. Synthesis of Berberine 9 Oxybutyl Derivative (B9OBU)

**Figure 13:**
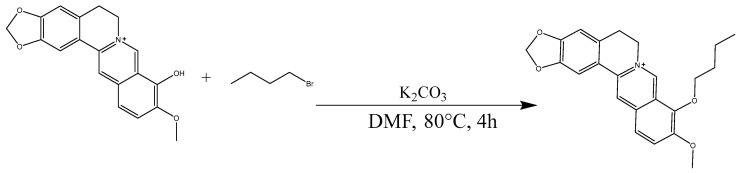
Synthesis of berberine 9 oxybutyl derivative (B9OBU).

The berberrubine (1 equivalents), n-butane bromide (3 equivalents), and potassium carbonate (3 equivalents) were dissolved in anhydrous DMF and stirred at a temperature of 80 °C for a duration of 4 h [20,21]. Following the removal of the solvent, purification was carried out using silica gel column chromatography to obtain a yellow solid derivative known as berberine 9 oxybutyl derivative (B9OBU) (Figure 13).

### 3.7. Determination of Minimum Inhibitory Concentrations 80 (MIC_80_)

The bacterial strains *Staphylococcus aureus* (*S. aureus*, 0485U), *Escherichia coli* (*E. coli*, 0335U), and *Pseudomonas aeruginosa* (*P. aeruginosa*, BNCC125486) were cultured in LB medium and incubated overnight at 37 °C in a shaking incubator. The culture was subsequently transferred to aseptic LB medium and incubated until reaching the logarithmic growth phase prior to utilization. The microdilution method was employed to determine the minimum inhibitory concentrations (MICs) of 9-berberrubine-(9→6′)-*O*-canagliflozin derivative (B9OC), berberine (BBR), canagliflozin (CAN), berberine 9 oxybutyl derivative (B9OBU), and a combination of berberine and canagliflozin (B + C) against *S. aureus*, *E. coli*, and *P. aeruginosa*. In brief, 95 µL of bacterial solution containing 5 × 10^5^ CFU/mL was mixed with 5 µL of B9OC, BBR, CAN, BBR + CAN, or B9OBU at various dilutions in a 96-well plate to achieve concentration gradients ranging from 0.01 mM to 5.12 mM for each drug; triplicates were performed for each concentration gradient. DMSO and dichloromethane were used as controls. The plate was then incubated at 37 °C for 24 h followed by measuring the OD_600_ using an enzyme-linked immunosorbent assay reader. MIC was defined as the drug concentration that exhibited antimicrobial activity equal to or above 80%.

### 3.8. Growth Curve

After the bacterial strains reached logarithmic growth phase, 95 µL of a bacterial solution (5 × 10^5^ CFU/mL) and 5 µL of B9OC, BBR, CAN, BBR + CAN, and B9OBU were added to a 96-well plate in order to achieve a concentration of 1/2 MIC for each drug. This process was repeated three times in parallel. A control group containing medium with 0.1% (*v*/*v*) DMSO and dichloromethane was also included. The plate was then incubated at 37 °C for 12 h and subsequently placed on a multifunctional microplate reader to measure OD_600_ at one-hour intervals over the course of 12 h.

### 3.9. Biofilm Growth

To investigate the antibacterial mechanism of the synthesized product B9OC, we determined its effect on bacterial biofilms. Bacterial cultures containing *S. aureus*, *E. coli*, and *P. aeruginosa* were adjusted to an optical density (OD_620_) of 0.1 and added to 96-well plates. The cultures were then treated with B9OC, BBR, CAN, BBR + CAN, and B9OBU at MIC and 1/2 MIC concentrations, respectively. After incubating at 37 °C for 24 h, the bacteria in the microplate were fixed with paraformaldehyde for 30 min. Following the removal of paraformaldehyde, the plate was dried at 55 °C before adding 200 μL of a 0.1% crystal violet dye which was left to stain at room temperature for 10 min. The wells were cleaned with sterile water to remove excess dye and then dried again at 55 °C. Subsequently, we added 200 μL of a solution containing glacial acetic acid (33%) which was left at 37 °C for another half hour to fully dissolve the attached crystal violet dye, finally measuring absorbance values at a wavelength of 570 nm using a microplate reader. Each group had three biological replicates set up as samples, and statistical analysis was performed to calculate any differences.

### 3.10. SDS-Polyacrylamide Gel Electrophoresis (SDS-PAGE)

SDS-PAGE was employed to analyze the impact of B9OC on *S. aureus* proteins. The preparation of SDS-PAGE gels followed the instructions provided by the kit manufacturer. *S. aureus* cells, treated with PBS and 1/2MIC of CAN, BBR, B + C, B9OBU, and B9OC for 24 h, were collected through centrifugation at 10,000× *g* for 5 min and subsequently washed twice with PBS. Protein loading buffer was added and boiled at 100 °C for 20 min; thereafter, the supernatant was obtained via centrifugation at 10,000× *g* for 5 min. The protein concentration was determined using the BCA Protein Concentration Assay kit (Beyotime, Shanghai, China) and adjusted uniformly across all samples to achieve a total loading volume of 10 μg. Following sample loading, a voltage of 60 V was applied for approximately 30–35 min before increasing it to 120 V for about an additional hour. Subsequently, Coomassie Brilliant blue ultrafast staining solution was used to decolorize the electrophoresed protein bands in order to isolate them.

### 3.11. Field Emission Scanning Electron Microscopy (FESEM)

*Staphylococcus aureus* at a concentration of 1 × 10^8^ CFU/mL was incubated with PBS and B9OC at half the minimum inhibitory concentration (MIC) for 24 h at 37 °C. The bacteria were then collected, fixed in 2.5% glutaraldehyde overnight at 4 °C, and washed three times with PBS. Subsequently, the bacterial precipitates were dehydrated using a series of ethanol concentrations (30%, 50%, 70%, 80%, 90%, 95%, and 100%) for 15 min each before being dried at room temperature. Finally, they were observed under a field emission scanning electron microscope (SU8010, Hitachi, Tokyo, Japan).

### 3.12. ADMET Prediction Based on Computer Aided Prediction

We utilized the iDrug platform (Tencent, Shenzhen, China), powered by cloud computing and artificial intelligence, to input compound B9OC and predict its ADMET properties.

### 3.13. Statistical Analysis

GraphPad Prism 9.0 software (GraphPad Software, San Diego, CA, USA) was used for the statistical analysis. Differences with statistical significance between groups were calculated by an ANOVA followed by Tukey’s post hoc test. *p* < 0.05 was considered statistically significant.

## 4. Conclusions

**Figure 14:**
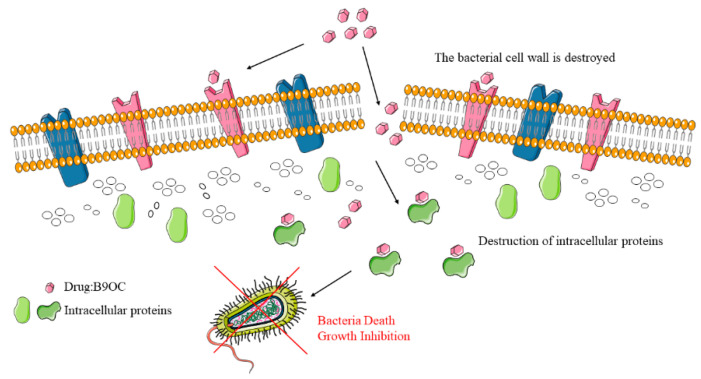
Bactericidal mechanism of compound B9OC [22].

This present study involved the initial pyrolysis of berberine to obtain berberrubine, followed by a classical Wilhelmson synthesis reaction where canagliflozin bromide was linked to berberine, resulting in the successful synthesis of the desired product B9OC. The minimal inhibitory concentration (MIC) of B9OC against the three bacteria demonstrated its superior antibacterial activity compared to berberine, while B9OBU exhibited lower antibacterial activity than berberine. Hence, it can be concluded that simply elongating the carbon chain length of C9 alone does not directly enhance the antibacterial activity of berberine. Compared to our previous research product BC, B9OC demonstrates enhanced bactericidal effects against *Staphylococcus aureus* and *Escherichia coli*. The findings from crystal violet staining, SDS-PAGE analysis, and FESEM observations indicate that B9OC possesses the ability to disrupt bacterial biofilms and target intracellular proteins. Moreover, it induces significant surface alterations such as wrinkling, indentation, and roughness on bacterial cell walls, ultimately leading to cell rupture (Figure 14). We employed computer-assisted prediction to evaluate the ADMET properties of compound B9OC. In the subsequent studies, we will further validate the pharmacokinetic parameters of compound B9OC through rigorous experimentation and analysis to ensure their accuracy and reliability.

## 5. Discussion

Research has demonstrated that diabetes can lead to compromised immune function, resulting in urinary tract infections being one of the major complications of the disease [23]. The primary pathogens responsible for these infections include *Staphylococcus aureus* and *Escherichia coli* [24]. Studies have indicated that diabetic patients use SGLT2 inhibitors such as canagliflozin, dapagliflozin, and empagliflozin are at an increased risk of developing genital infections compared to those using placebo or other active treatments [25,26]. In recent years, many countries and regions have been facing the challenge of an aging population, with diabetes emerging as one of the survival risks for elderly individuals. Furthermore, research has also shown that antimicrobial resistance among common pathogens plays a crucial role in reducing mortality rates among elderly patients with urinary tract infections [27,28]. Therefore, our study on synthesizing a novel compound B9OC holds significant promise in addressing both diabetes-related and non-diabetes-related factors contributing to urinary tract infections. Additionally, it aims to tackle genital infections resulting from SGLT2 inhibitor usage and bacterial resistance issues. In conclusion, B9OC demonstrates remarkable efficacy as a berberine derivative exhibiting broad-spectrum antibacterial activity against both Gram-positive and Gram-negative bacteria. This compound holds immense potential for diverse applications and presents a novel solution to the pressing issue of bacterial resistance.

## Figures and Tables

**Table 1:** Minimum inhibitory concentrations (MIC80) of B9OC, BBR, BBR + CAN, and B9OBU against different strains.

Bacterial Strain	Drug	MIC_80_ (mM)	MIC_80_ (mM) 95% CI
	B9OC	0.035 **^,#,$$^	0.029~0.042
*Staphylococcus aureus*	BBR	0.188	0.164~0.218
0485U	BBR + CAN	0.063	0.049~0.085
	B9OBU	0.591	0.501~0.707
	CAN	2.377	2.076~2.749
	BC	0.380	0.320~0.440
	B9OC	0.285 **^,##,$$^	0.239~0.343
*Escherichia coli*	BBR	0.982	0.828~1.174
0335U	BBR + CAN	0.435	0.367~0.529
	B9OBU	2.241	1.952~2.539
	CAN	1.876	1.614~2.201
	BC	0.390	0.350~0.430
	B9OC	0.331 ^##,$$^	0.300~0.367
*Pseudomonas aeruginosa*	BBR	0.665	0.575~0.781
BNCC125486	BBR + CAN	0.251	0.221~0.289
	B9OBU	1.283	1.204~1.349
	CAN	NI	NI
	BC	0.220	0.180~0.260

95% CI: 95% confidence intervals. ** *p* < 0.01 vs. BBR; # *p* < 0.05, ## *p* < 0.01 vs. BBR + CAN; $$ *p* < 0.01 vs. B9OBU.

**Table 2:** ADMET prediction of B9OC based on computer-aided prediction.

Absorption Properties		Metabolism Properties		Distribution Properties	
Thermodynamic Solubility, Log (S, mol/L)	−3.09	HLM-CLint (μL/min/mg)	67.214	Plasma Protein Binding(human)	0.97
ESOL_Kinetic, Log (S, mol/L)	−4.42	MLM-CLint (μL/min/mg)	415.94	Blood–Brain barrier Permeability (BBBP) Probability	0.26
Pampa	4.85	RLM-CLint (μL/min/mg)	89.628	Blood–Brain Ratio	−0.62
MDCK	3.43	CYP Induction Probability	0.269		
Caco-2 Permeability (10^−6^, apical to basolateral)	3.39	CYP Inhibition Probability (1A2)	0.48	**Excretion Properties**	
P-gp Substrate Probability	0.96	CYP Inhibition Probability (2C19)	0.899	Human Clearance	0.401
P-gp Inhibition Probability	0.55	CYP Inhibition Probability (2C9)	0.931		
Human Intestinal Absorption (HIA) Probability	0.15	CYP Inhibition Probability (2D6)	0.76		
Oral Bioavailability (human)	0.47	CYP Inhibition Probability (3A4)	0.589		
**Toxicity Properties**		hERG Inhibition Probability	0.124		
		hERG Inhibition probability_cls10	0.614	Phospholipidosis	0.771
		hERG Inhibition probability_cls50	0.998	Reproductive Toxicity	0.997
		Ames Toxicity Probability	0.319	NR-AR	0.032
		Hek293 Toxicity Probability	0.952	NR-AR-LBD	0.048
		Hepatic Toxicity Probability	0.967	NR-AhR	0.325
		Eye Corrosion	0	NR-Aromatase	0.369
		Log (LD50)	2.915	NR-ER	0.144
		Phototoxicity	0.259	NR-ER-LBD	0.066
		Tubulin Inhibition	1	NR-PPAR-gamma	0.119
		Eye Irritation	0	SR-ARE	0.613
		DILI	0.928	SR-ATAD5	0.073
		Genotoxicity	0.99	SR-HSE	0.244
		Carcinogenicity	0.227	SR-MMP	0.688
		Mutagenicity	0.001	SR-p53	0.431

Powered by https://drug.ai.tencent.com (accessed on 7 December 2023).

## Data Availability

All data are available in a repository or online in accordance with funder data retention policies.

## Supplementary Materials

The following supporting information can be downloaded at: https://www.mdpi.com/article/10.3390/molecules29010273/s1, Figures S1–S3: ^1^H, ^13^C NMR and MS spectra of compound B9OC; Figure S4: The purity of B9OC from HPLC; Figures S5 and S6: ^1^H, ^13^C NMR spectra of compound Berberrubine; Figures S7 and S8: ^1^H, ^13^C NMR spectra of compound Canagliflozin bromide; Figures S9 and S10: ^1^H, ^13^C NMR spectra of compound B9OBU; Figures S11 and S12: ^1^H, ^13^C NMR spectra of compound BBR; Figure S13: Western blot images for PBS-B9OC; Figure S14: Western blot images for PBS-BBR-CAN-BBR + CAN; Figure S15: ADMET Prediction of B9OC Based on Computer Aided.

## References

[1] Song D., Hao J., Fan D. (2020). Biological properties and clinical applications of berberine. Front. Med..

[2] Kong W.J., Vernieri C., Foiani M., Jiang J.D. (2020). Berberine in the treatment of metabolism-related chronic diseases: A drug cloud (dCloud) effect to target multifactorial disorders. Pharmacol. Ther..

[3] Akbar M., Shabbir A., Rehman K., Akash M.S.H., Shah M.A. (2021). Neuroprotective potential of berberine in modulating Alzheimer’s disease via multiple signaling pathways. J. Food Biochem..

[4] Cao R.Y., Zheng Y., Zhang Y., Jiang L., Li Q., Sun W., Gu W., Cao W., Zhou L., Zheng H. (2021). Berberine on the Prevention and Management of Cardiometabolic Disease: Clinical Applications and Mechanisms of Action. Am. J. Chin. Med..

[5] Rauf A., Abu-Izneid T., Khalil A.A., Imran M., Shah Z.A., Emran T.B., Mitra S., Khan Z., Alhumaydhi F.A., Aljohani A.S.M. (2021). Berberine as a Potential Anticancer Agent: A Comprehensive Review. Molecules.

[6] Tian C.M., Jiang X., Ouyang X.X., Zhang Y.O., Xie W.D. (2016). Berberine enhances antidiabetic effects and attenuates untoward effects of canagliflozin in streptozotocin-induced diabetic mice. Chin. J. Nat. Med..

[7] Hao W., Che S., Li J., Luo J., Zhang W., Chen Y., Zhao Z., Wei H., Xie W. (2022). Synthesis of Berberine and Canagliflozin Chimera and Investigation into New Antibacterial Activity and Mechanisms. Molecules.

[8] Cui X., Lü Y., Yue C. (2021). Development and Research Progress of Anti-Drug Resistant Bacteria Drugs. Infect. Drug Resist..

[9] Domingues C.P.F., Rebelo J.S., Dionisio F., Nogueira T. (2023). Multi-Drug Resistance in Bacterial Genomes—A Comprehensive Bioinformatic Analysis. Int. J. Mol. Sci..

[10] Jamshaid F., Dai J., Yang L.X. (2020). New Development of Novel Berberine Derivatives against Bacteria. Mini Rev. Med. Chem..

[11] Wang J., Yang T., Chen H., Xu Y.N., Yu L.F., Liu T., Tang J., Yi Z., Yang C.G., Xue W. (2017). The synthesis and antistaphylococcal activity of 9,13-disubstituted berberine derivatives. Eur. J. Med. Chem..

[12] Yao L., Wu L.L., Li Q., Hu Q.M., Zhang S.Y., Liu K., Jiang J.Q. (2018). Novel berberine derivatives: Design, synthesis, antimicrobial effects, and molecular docking studies. Chin. J. Nat. Med..

[13] Xiao D., Liu Z., Zhang S., Zhou M., He F., Zou M., Peng J., Xie X., Liu Y., Peng D. (2018). Berberine Derivatives with Different Pharmacological Activities via Structural Modifications. Mini Rev. Med. Chem..

[14] Liu Y., Long S., Zhang S., Tan Y., Wang T., Wu Y., Jiang T., Liu X., Peng D., Liu Z. (2021). Synthesis and antioxidant activities of berberine 9-O-benzoic acid derivatives. RSC Adv..

[15] Cao H., Liao S., Zhong W., Xiao X., Zhu J., Li W., Wu X., Feng Y. (2017). Synthesis, Characterization, and Biological Evaluations of 1,3,5-Triazine Derivatives of Metformin Cyclization with Berberine and Magnolol in the Presence of Sodium Methylate. Molecules.

[16] Li R., Wu J., He Y., Hai L., Wu Y. (2014). Synthesis and in vitro evaluation of 12-(substituted aminomethyl) berberrubine derivatives as anti-diabetics. Bioorg Med. Chem. Lett..

[17] Wang Y.X., Yang L., Wang H.Q., Zhao X.Q., Liu T., Li Y.H., Zeng Q.X., Li Y.H., Song D.Q. (2018). Synthesis and Evolution of Berberine Derivatives as a New Class of Antiviral Agents against Enterovirus 71 through the MEK/ERK Pathway and Autophagy. Molecules.

[18] Sobolova K., Hrabinova M., Hepnarova V., Kucera T., Kobrlova T., Benkova M., Janockova J., Dolezal R., Prchal L., Benek O. (2020). Discovery of novel berberine derivatives with balanced cholinesterase and prolyl oligopeptidase inhibition profile. Eur. J. Med. Chem..

[19] Raghuvanshi R., Jamwal A., Nandi U., Bharate S.B. (2023). Multitargeted C9-substituted ester and ether derivatives of berberrubine for Alzheimer’s disease: Design, synthesis, biological evaluation, metabolic stability, and pharmacokinetics. Drug Dev. Res..

[20] Lin H.J., Ho J.H., Tsai L.C., Yang F.Y., Yang L.L., Kuo C.D., Chen L.G., Liu Y.W., Wu J.Y. (2020). Synthesis and In Vitro Photocytotoxicity of 9-/13-Lipophilic Substituted Berberine Derivatives as Potential Anticancer Agents. Molecules.

[21] Long Y.H., Bai L.P., Qin Y., Pang J.Y., Chen W.H., Cai Z., Xu Z.L., Jiang Z.H. (2006). Spacer length and attaching position-dependent binding of synthesized protoberberine dimers to double-stranded DNA. Bioorg Med. Chem..

[22] Servier Medical Art by Servier is licensed under a Creative Commons Attribution 3.0 Unported License. https://smart.servier.com.

[23] Kamei J., Yamamoto S. (2021). Complicated urinary tract infections with diabetes mellitus. J. Infect. Chemother..

[24] Neugent M.L., Hulyalkar N.V., Nguyen V.H., Zimmern P.E., De Nisco N.J. (2020). Advances in Understanding the Human Urinary Microbiome and Its Potential Role in Urinary Tract Infection. mBio.

[25] Li D., Wang T., Shen S., Fang Z., Dong Y., Tang H. (2017). Urinary tract and genital infections in patients with type 2 diabetes treated with sodium-glucose co-transporter 2 inhibitors: A meta-analysis of randomized controlled trials. Diabetes Obes. Metab..

[26] Lega I.C., Bronskill S.E., Campitelli M.A., Guan J., Stall N.M., Lam K., McCarthy L.M., Gruneir A., Rochon P.A. (2019). Sodium glucose cotransporter 2 inhibitors and risk of genital mycotic and urinary tract infection: A population-based study of older women and men with diabetes. Diabetes Obes. Metab..

[27] Kakde P., Redkar N.N., Yelale A. (2018). Urinary Tract Infection in Elderly: Clinical Profile and Outcome. J. Assoc. Physicians India.

[28] Akash M.S.H., Rehman K., Fiayyaz F., Sabir S., Khurshid M. (2020). Diabetes-associated infections: Development of antimicrobial resistance and possible treatment strategies. Arch. Microbiol..

