# Supplementary material for: Synthesis of New Derivatives of Berberine Canagliflozin and Study of Their Antibacterial Activity and Mechanism
Source: Molecules. 2024 Jan 4;29(1):273. doi: 10.3390/molecules29010273 (PMC10780567; doi:10.3390/molecules29010273)
Supplement: Supplementary file 1 [file molecules-29-00273-s001.zip › molecules-2766484-supplementary.pdf]

Supporting Information For

Synthesis of new derivatives of berberine canagliflozin and study on their antibacterial activity and mechanism

Jinsheng Li [1,2,3], Xueli Hou [1,2,3], Jinlong Xiao [2,3], Li Zhu [2,3], Yujie Deng [2,3], Ziyi Li [2,3], Zijian Zhao [1,2,3,*], Zhenghong Luo [2,3,*] and Hao Wei [1,2]

[1] Department of Pharmacology, Shaanxi University of Chinese Medicine, Xianyang 712046, China; jsli1006@163.com (J.L.); xlhou199885@163.com (X.H.); weihao217@163.com (H.W.)

[2] School of Chemistry and Materials Science, Huaihua University, Huaihua 418000, China; jlxiao0204@163.com (J.X.); lizzz082803@163.com (L.Z.); dyj20040128@163.com (Y.D.); zyli1202@163.com (Z.L.);

[3] Key Laboratory of Research and Utilization of Ethnomedicinal Plant Resources of Hunan Province, Huaihua University, Huaihua 418000, China

* Correspondence: luozhenghong06@163.com (Z.L.); zzj@hhtc.edu.cn (Z.Z.)

CATALOG

Figure S 1 ^{1}H NMR spectra of compound B9OC 2

Figure S 2 ^{13}C NMR spectra of compound B9OC 2

Figure S 3 MS spectra of compound B9OC 3

Figure S 4 The purity of B9OC from HPLC 3

Figure S 5 ^{1}H NMR spectra of compound Berberrubine 4

Figure S 6 ^{13}C NMR spectra of compound Berberrubine 4

Figure S 7 ^{1}H NMR spectra of compound Canagliflozin bromide 5

Figure S 8 ^{13}C NMR spectra of compound Canagliflozin bromide 5

Figure S 9 ^{1}H NMR spectra of compound B9OBU 6

Figure S 10 ^{13}C NMR spectra of compound B9OBU 6

Figure S 11 ^{1}H NMR spectra of compound BBR 7

Figure S 12 ^{13}C NMR spectra of compound BBR........ 7

Figure S 13 Western blot images for PBS-B9OC 8

Figure S 14 Western blot images for PBS-BBR-CAN-BBR+CAN 8

Figure S 15 ADMET Prediction of B9OC Based on Computer Aided 9

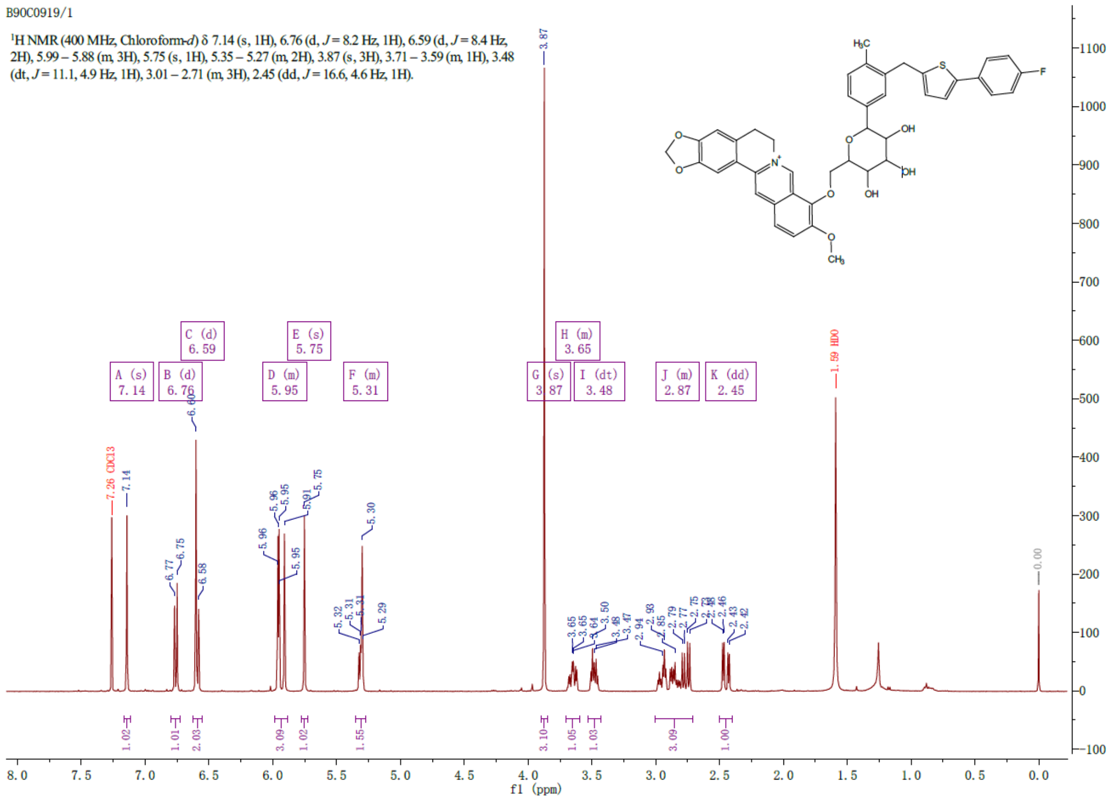

Figure S 1 ^{1}H NMR spectra of compound **B9OC**

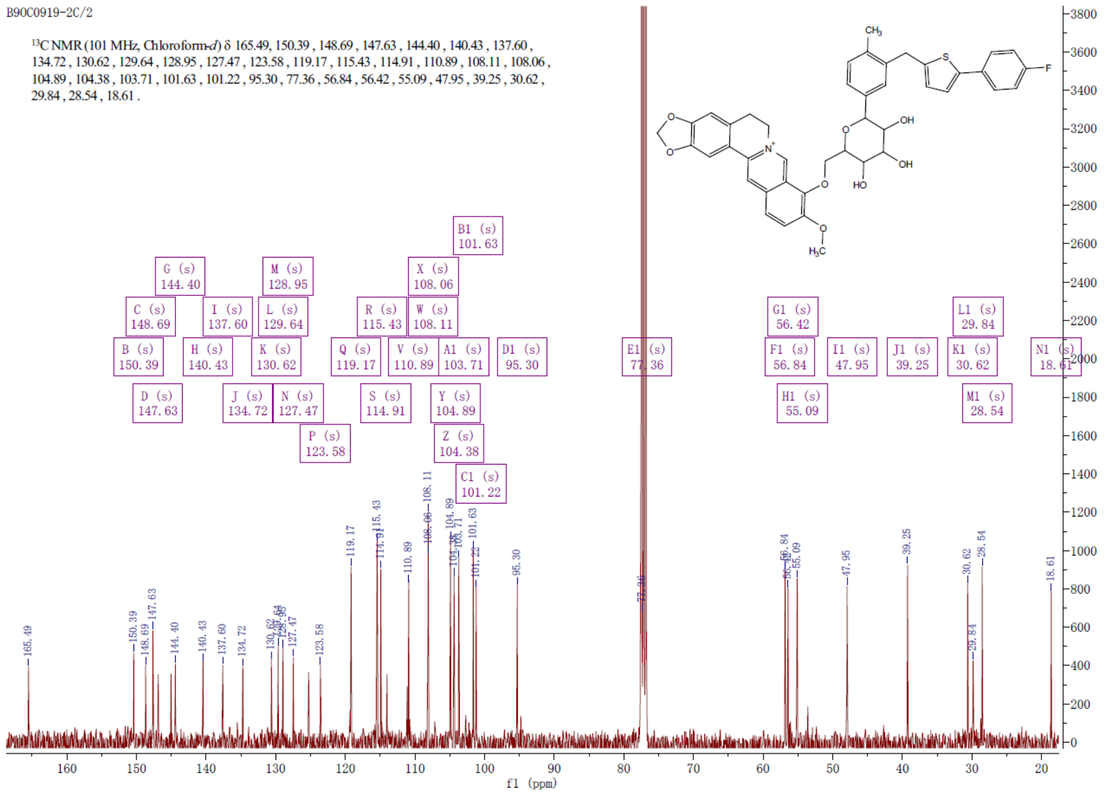

Figure S 2 ^{13}C NMR spectra of compound **B9OC**

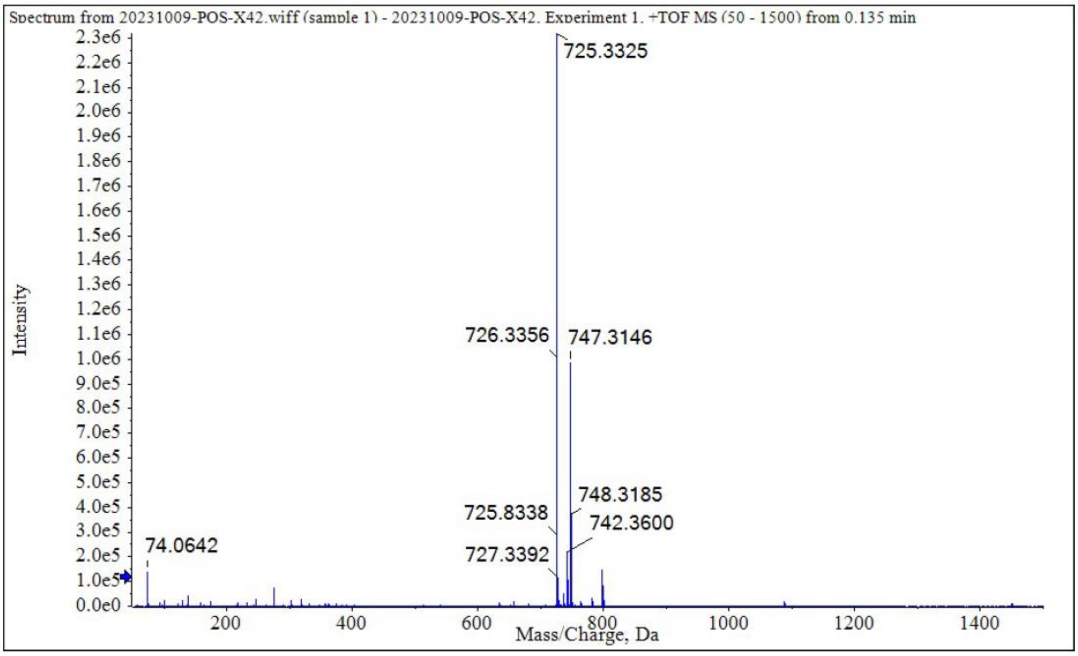

Figure S 3 MS spectra of compound B9OC

SHIMADZU LabSolutions **Analysis report**

<Sample Information>

Sample	: b9oca365nm
Sample ID	: 01
Inj. Volume	: 10 uL
Acquisition Date	: 2023/9/13 10:37:25

<Chromatogram>

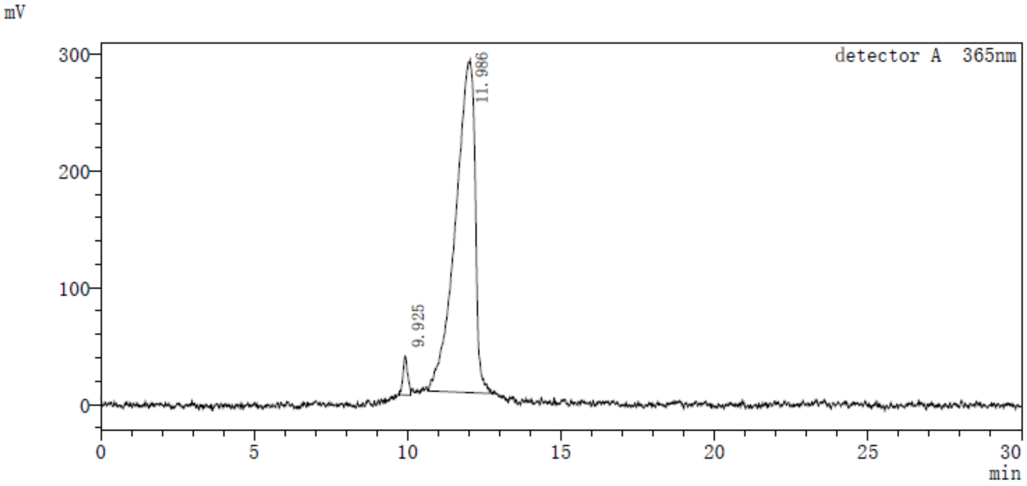

<Chromatogram peak table>

detector A 365nm

number	retention time	peak area	hight	concentration
1	9.925	353117	32886	2.743 %
2	11.986	12519188	282176	97.257 %
总计		12872305	315063	

Figure S 4 The purity of B9OC from HPLC

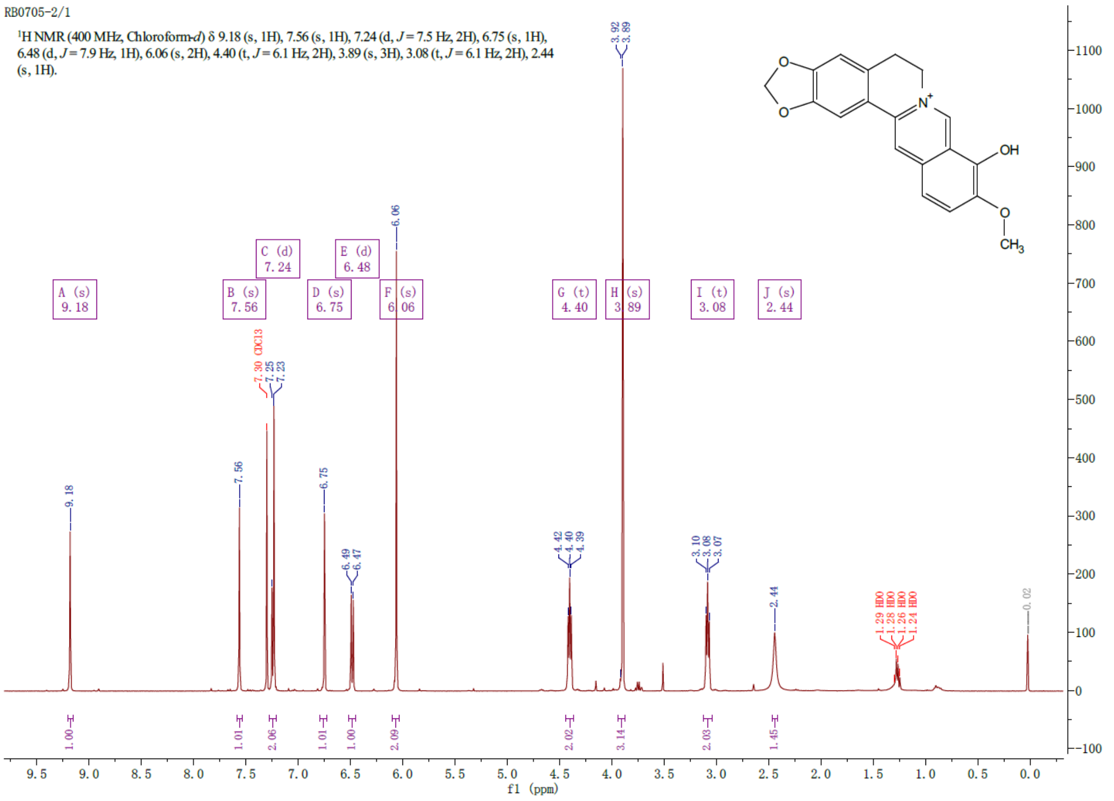

Figure S 5 ^{1}H NMR spectra of compound **Berberrubine**

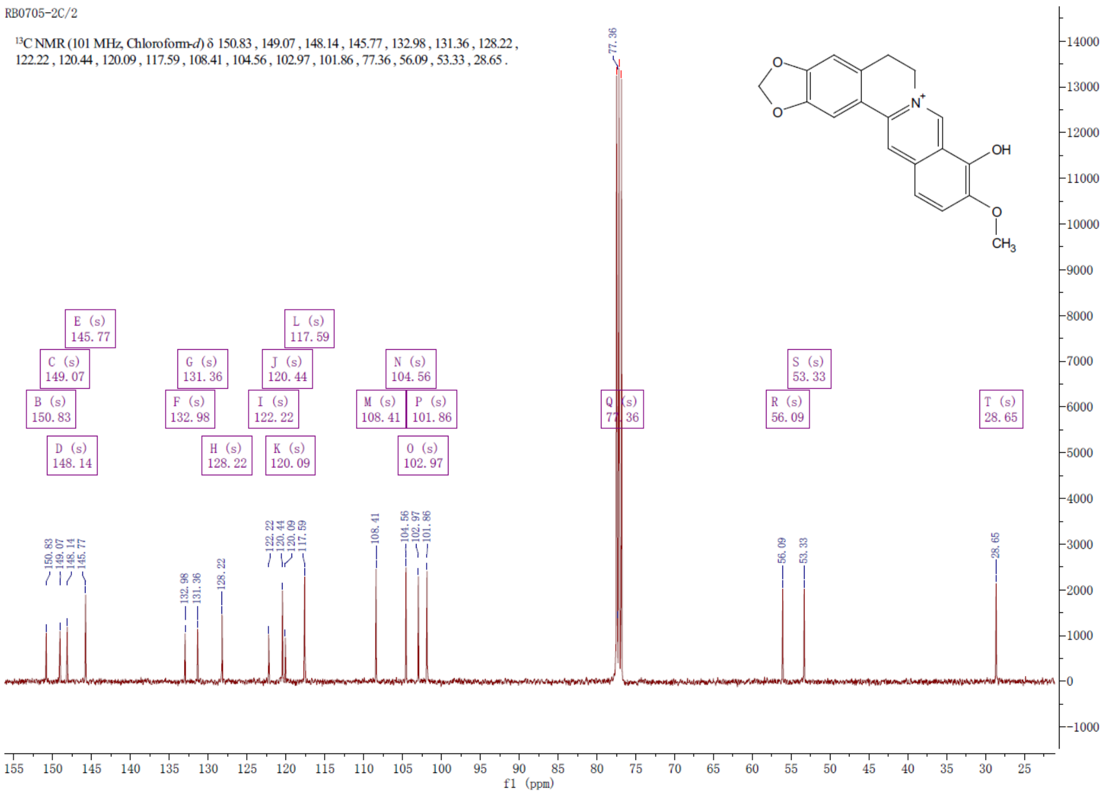

Figure S 6 ^{13}C NMR spectra of compound **Berberrubine**

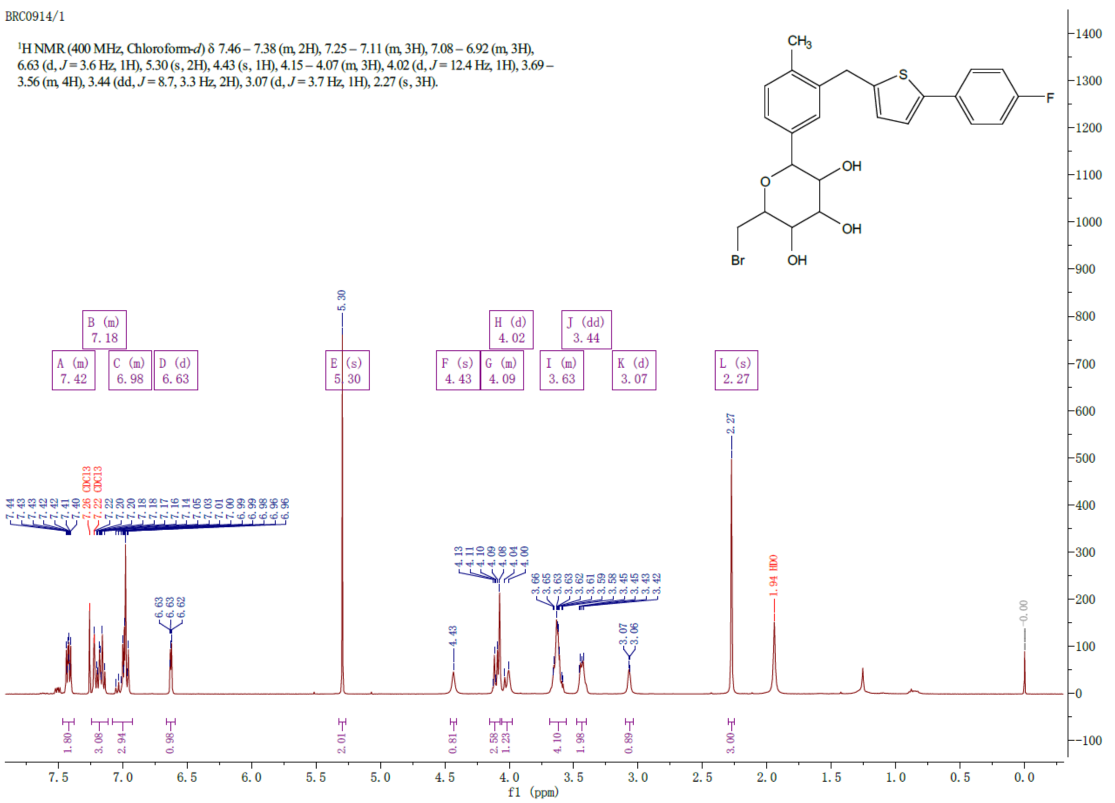

Figure S 7 ^{1}H NMR spectra of compound **Canagliflozin bromide**

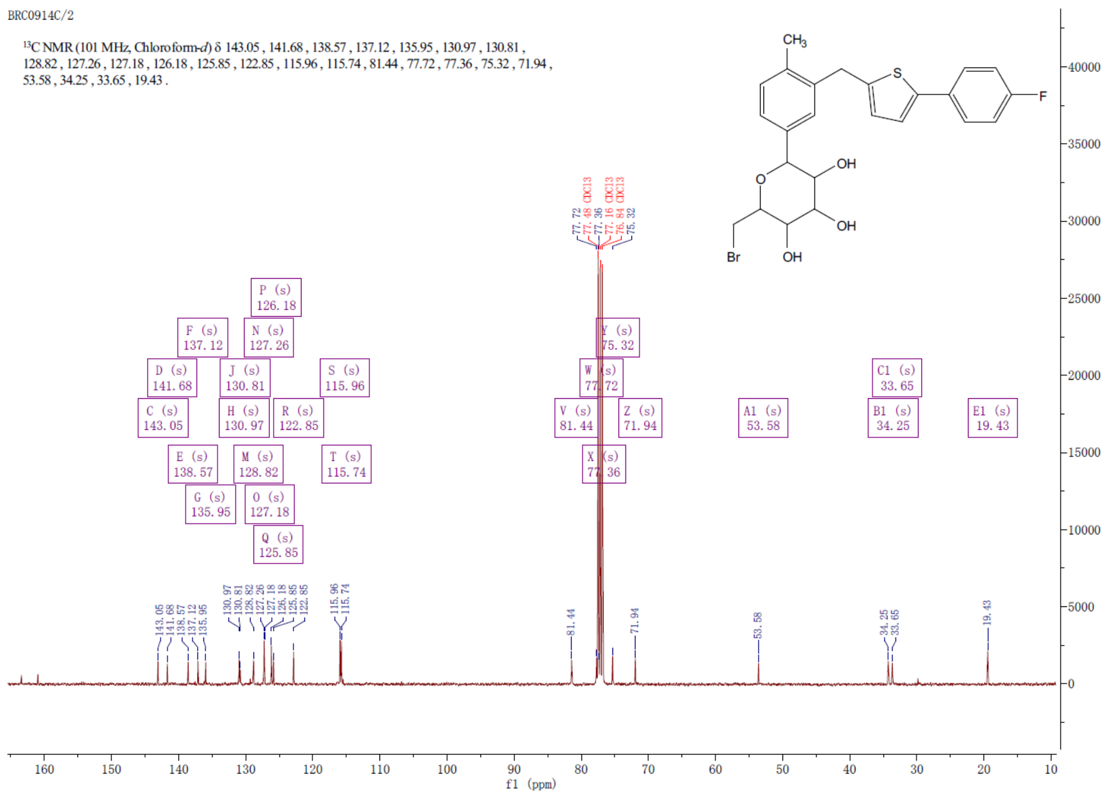

Figure S 8 ^{13}C NMR spectra of compound **Canagliflozin bromide**

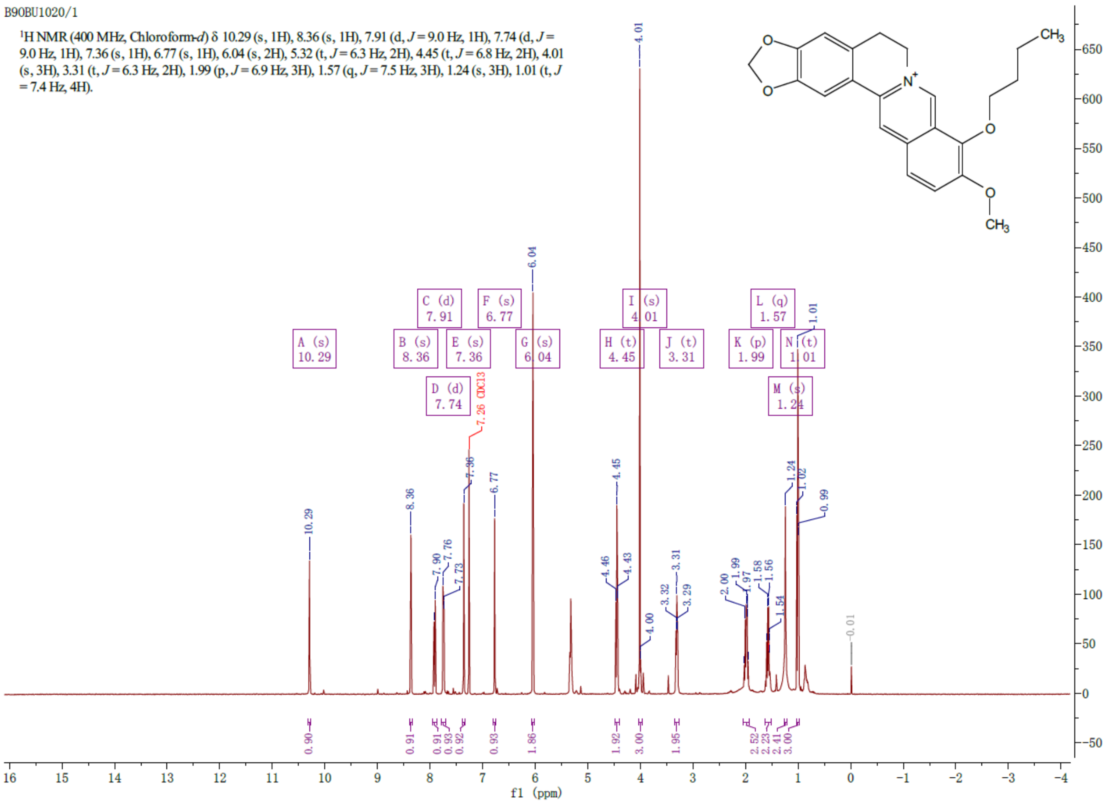

Figure S 9 ¹H NMR spectra of compound **B9OBU**

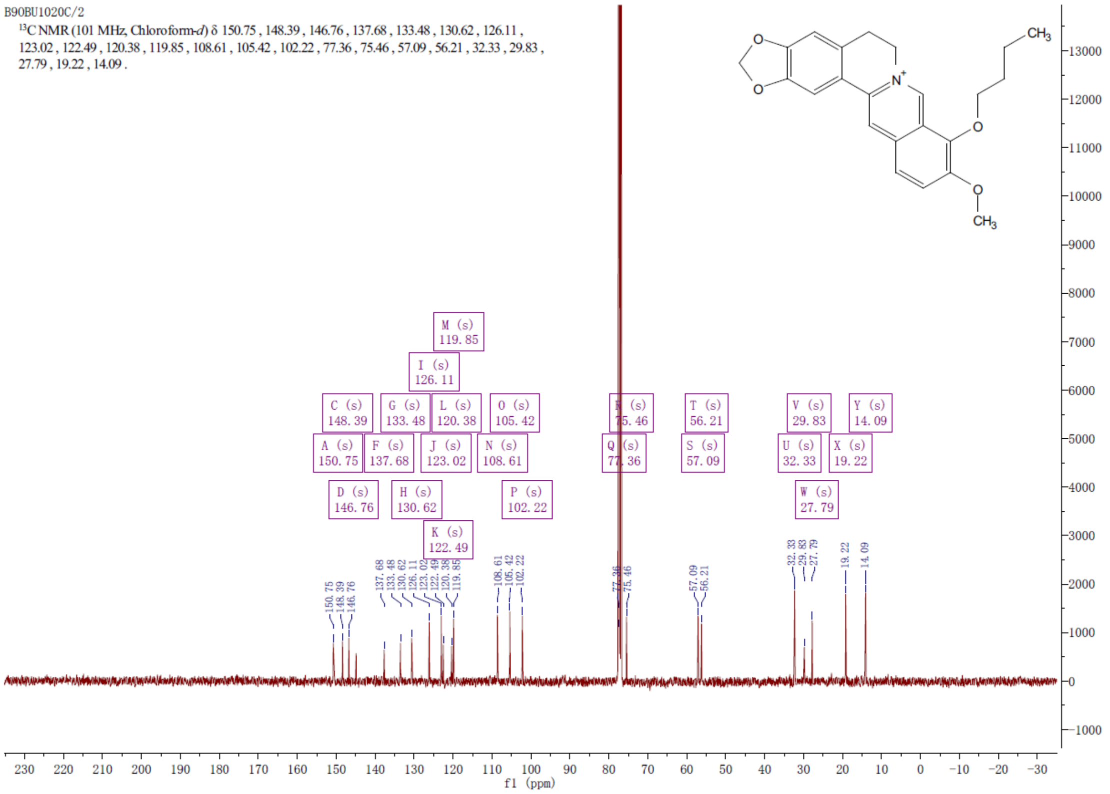

Figure S 10 ¹³C NMR spectra of compound **B9OBU**

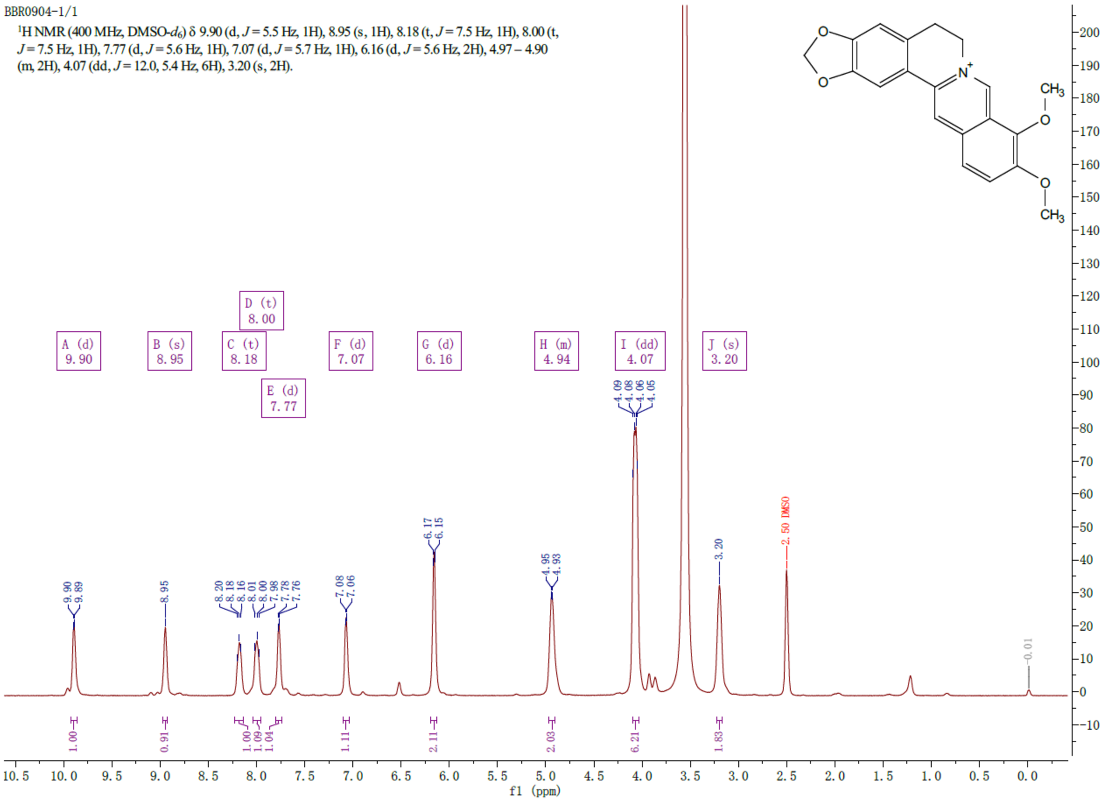

Figure S 11 ^{1}H NMR spectra of compound **BBR**

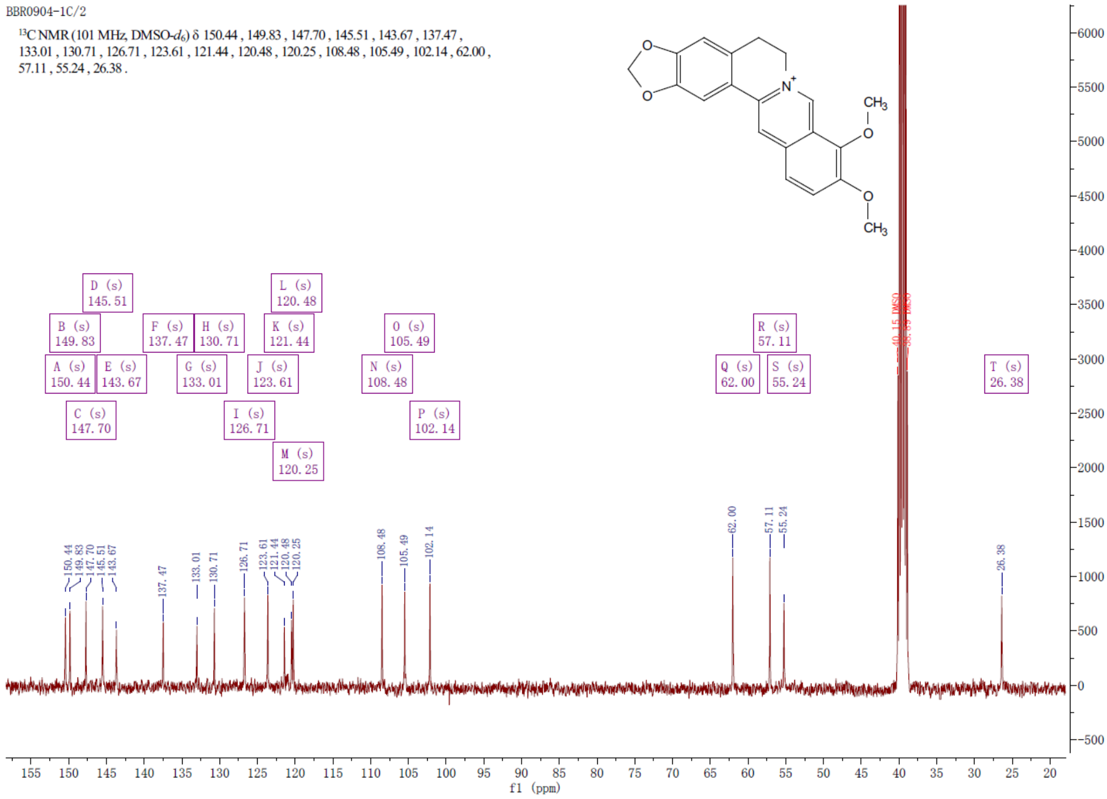

Figure S 12 ^{13}C NMR spectra of compound **BBR**

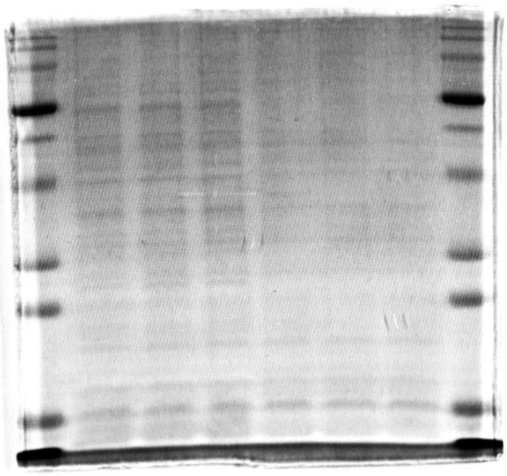

Figure S 13 **Western blot images for PBS-B9OC**

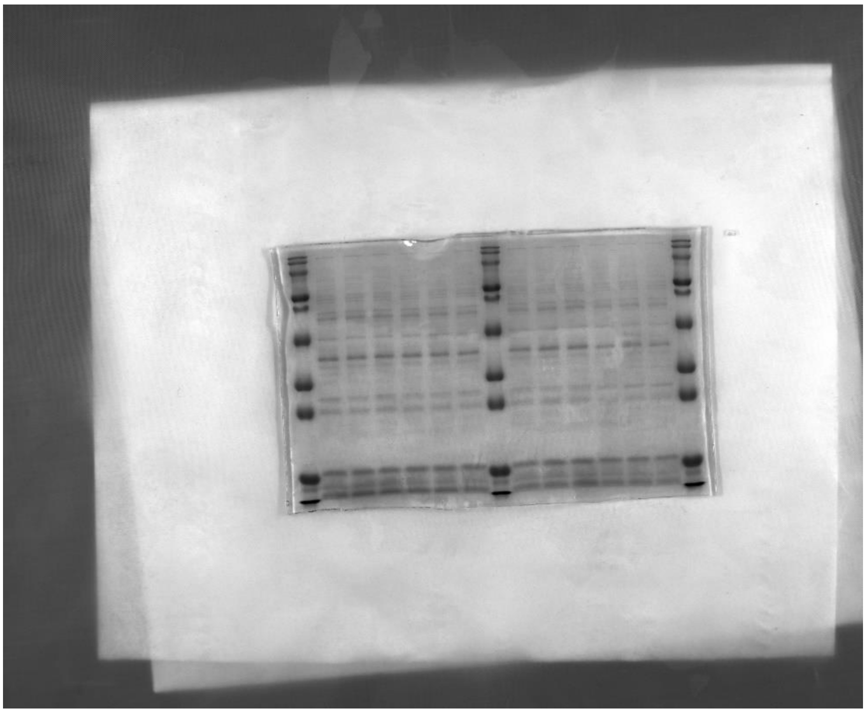

Figure S 14 **Western blot images for PBS-BBR-CAN-BBR+CAN**

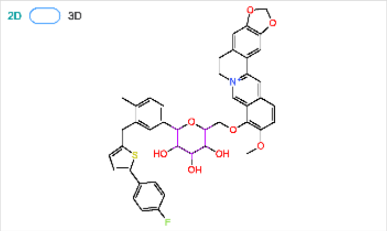

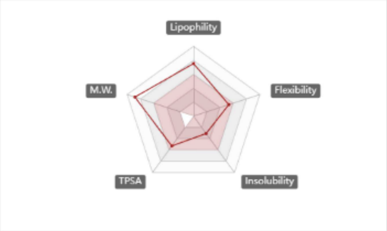

Basic Properties

Formula	$C_{43}H_{39}FNO_6S^+$
Molecular Weight	748.85
Fraction Csp3	0.28
Num. of Heavy Atoms	54
Num. Rotatable Bonds	8
Num. H-bond Acceptors	9
Num. H-bond Donors	3
TPSA	110.72
CLogP	6.46
Ring Count	9
Aromatic Ring Count	6
PAINS	0 alert
LogD	3.23
pKa	
Strongest Acidic pKa	7.19
Strongest Basic pKa	n./a.

Absorption Properties

Solubility	
Thermodynamic Solubility, Log(S, mol/L)	-3.09
ESOL_Kinetic, Log(S, mol/L)	-4.42
Solubility_ds1	0.95
Solubility_ds10	0.91
Solubility_ds100	0.86
Pampa	4.85
MDCK	3.43
Caco-2 Permeability(10e-6, apical to basolat…	3.39
P-gp Substrate Probability	0.96
P-gp Inhibition Probability	0.55
Human Intestinal Absorption(HIA) Probability	0.15
Oral Bioavailability(human)	47%

Distribution Properties

Plasma Protein Binding(human)	97%
Blood-Brain Barrier Permeability (BBBP) Pro…	0.26
Blood Brain Ratio	-0.62

Metabolism Properties

HLM-CLint (μL/min/mg)	67.21
MLM-CLint (μL/min/mg)	415.94
RLM-CLint (μL/min/mg)	89.63
CYP Induction Probability	0.27
CYP Inhibition Probability	
1A2	0.48
2C19	0.9
2C9	0.93
2D6	0.76
3A4	0.59

Excretion Properties

Human Clearance	0.4

Toxicity Properties

hERG Inhibition Probability	0.12
hERG Inhibition probability_cls10	0.61
hERG Inhibition probability_cls50	1
Ames Toxicity Probability	0.32
Hek293 Toxicity Probability	0.95
Hepatic Toxicity Probability	0.97
Eye Corrosion	0
Log(LD50)	2.91
Phototoxicity	0.26
Tubulin Inhibition	1
Eye Irritation	0
DILI	0.93
Genotoxicity	0.99
Carcinogenicity	0.23
Mutagenicity	0
Phospholipidosis	0.77
Reproductive Toxicity	1
TOX21 Probability	
NR-AR	0.33
NR-AR-LBD	0.35
NR-AhR	0.33
NR-Aromatase	0.37
NR-ER	0.14
NR-ER-LBD	0.37
NR-PPAR-gamma	0.12
SR-ARE	0.81
SR-ATAD5	0.37
SR-HSE	0.24
SR-MMP	0.69
SR-p53	0.43

Figure S 15 ADMET Prediction of B9OC Based on Computer Aided
